# A poly-herbal blend (Herbagut®) on adults presenting with gastrointestinal complaints: a randomised, double-blind, placebo-controlled study

**DOI:** 10.1186/s12906-018-2168-y

**Published:** 2018-03-20

**Authors:** Adrian L. Lopresti, Hemant Gupta, Stephen J. Smith

**Affiliations:** 1 School of Psychology and Exercise Science, Murdoch University, Perth, WA 6150 Australia; 2 Samvedna Hospital, B-27 88G, Ravindrapuri, New Colony, Varanasi, U.P 221005 India; 3 Southern Cross University, Military Road, East Lismore, NSW 2480 Australia

**Keywords:** Gastrointestinal function, Digestion, Herbal treatment, Constipation, Clinical trial

## Abstract

**Background:**

To evaluate the efficacy and tolerability of a poly-herbal formulation, Herbagut, for the treatment of gastrointestinal symptoms and its effect on quality of life parameters in patients presenting with self-reported, unsatisfactory bowel habits.

**Methods:**

This was a randomised, double-blind, placebo-controlled trial. Fifty adults with self-reported unsatisfactory bowel habits, primarily characterised by chronic constipation were randomly allocated to take Herbagut or a matching placebo for 28 days. Efficacy of gastrointestinal changes was measured by the completion of a patient daily diary evaluating changes in stool type (Bristol Stool Form Scale), ease of bowel movements, and feeling of complete evacuation; and the Gastrointestinal Symptom Rating Scale (GSRS). Changes in quality of life were also examined using the World Health Organization Quality of Life – abbreviated version (WHOQOL-BREF), and the Patient Assessment of Constipation-Quality of Life (PAC-QOL).

**Results:**

All participants completed the 28-day trial with no adverse events reported. Compared to the placebo, weekly bowel movements increased over time (*p* < .001), as did self-reported, normal bowel motions (76% vs 4%; *p* < .001). Self-reported incomplete evacuation was also lower in the Herbagut group compared to placebo (24% vs 76%; *p* = <.001). GSRS domain ratings for abdominal pain, constipation, diarrhoea, indigestion, and reflux also decreased significantly in people taking Herbagut compared to placebo (*p* < .001, for all domains). Moreover, quality of life significantly improved in the Herbagut group compared to placebo as indicated by significantly greater improvement in WHOQOL-BREF domain ratings for overall quality of life, social relations, environmental health, psychological health, and physical health (*p* < .001, for all domains); and PAC-QOL domain ratings for physical discomfort, psychosocial discomfort, worries and concerns, and life satisfaction (*p* < .001, for all domains). The changes were considered clinically meaningful as evidenced by their large effect sizes.

**Conclusion:**

Herbagut ingestion over a 28-day period resulted in improvements in several gastrointestinal symptoms and overall quality of life. Further investigation utilising larger sample sizes and diverse clinical and cultural populations are needed.

**Trial registration:**

Clinical Trials Registry- India /2016/11/007479. Registered 24 April 2015 (retrospectively registered).

**Electronic supplementary material:**

The online version of this article (10.1186/s12906-018-2168-y) contains supplementary material, which is available to authorized users.

## Background

Functional gastrointestinal disorders (FGIDs) are a group of conditions with no identifiable structural or biochemical abnormality. They are distinguished by a range of symptoms including motility disturbance, visceral hypersensitivity, disturbed mucosal and immune function, altered gut microbiota, and altered central nervous system processing. Based on the Rome IV classification system, there are 33 FGIDs in adults and 20 paediatric disorders, all of which are theorised to result from disturbances in gut-brain interactions [1].

FGIDs are associated with a reduced quality of life, characterised by impairments in social, mental, occupational, and physical function [2, 3]. Approximately 30% of adults in primary care settings are believed to suffer from depression, and between 30 to 50% have a comorbid anxiety disorder [4, 5]. People with FGIDs also have an increased non-gastrointestinal (GI) medical burden characterised by a greater number of non-GI diagnoses, medical consultations, and pharmaceutical prescriptions compared to GI symptom-free adults [6]. Chronic constipation is particularly problematic as it is estimated to affect up to 27% of the world’s population, with greater prevalence in women and adults over the age of 65 years [7]. Results from a US population-based cohort study with over 30,000 person-years of follow-up confirmed that chronic constipation was associated with poorer survival rates over an approximate 20-year period [8].

Constipation is a symptom-based disorder based on subjective patient descriptions. Consequently, there is often a lack of agreement between physicians, patients, and researchers regarding its defining characteristics. However, this has been improved by the increased adoption of the Rome diagnostic criteria. Based on the Rome IV criteria, chronic constipation must include two or more of the following: (a) straining during at least 25% of defecations, (b) lumpy or hard stools in at least 25% of defecations, (c) sensation of incomplete evacuation for at least 25% of defecations, (d) sensation of anorectal obstruction/blockage for at least 25% of defecations, (e) manual manoeuvres to facilitate at least 25% of defecations (e.g., digital evacuation, support of the pelvic floor), and (f) fewer than three spontaneous defecations per week. In addition, loose stools are rarely present without the use of laxatives, and there are insufficient criteria to meet the diagnosis of irritable bowel syndrome [9]. These symptoms must be present for at least 6 months before the diagnosis and should be present for at least the past 3 months.

Treatment options for constipation typically comprise recommendations of increased fluid intake, greater consumption of high-fibre-containing foods, increased physical activity, fibre supplementation, prescription of osmotic or stimulant laxatives, and possible pelvic floor therapy [10]. Identifying and modifying potential contributory or causative influences are also recommended including a review of medication use, psychiatric presentation, endocrine and metabolic disorders (e.g., diabetes mellitus, hyperparathyroidism, hypothyroidism, and chronic renal failure), and neurological disorders (e.g., dementia, Parkinson disease, neuropathies, multiple sclerosis, and spinal cord injuries) [11–13].

Complementary and alternative medicine (CAM) is commonly used by adults with GI conditions. An interview of over 13,000 US citizens with GI conditions confirmed that 42% used CAM in the past year (although only 3% of respondents used CAM for the treatment of GI issues), with herbs and supplements the most frequently used CAM modality [14]. In another study, 44 % of adults attending outpatient GI clinics also reported using CAM therapies [15]. Given this high adoption, investigations into CAM therapies are essential to ensure their safety and efficacy for individuals with GI disorders.

Natural plant compounds present as potential options to restore GI health and reduce GI symptoms. For example, there is mounting evidence from preclinical and clinical evidence that many natural compounds have anti-inflammatory and immunoregulatory influences, reduce oxidative stress, modulate intracellular signalling transduction pathways, and can alter gut microbiota [16]. Altering these molecular mechanisms can have beneficial effects on GI function. Moreover, plant-derived natural compounds also have the potential to lower visceral hypersensitivity (a decreased threshold of stimuli perception generated from the GI tract), which is a common problem in FGIDs such as irritable bowel syndrome [17].

### Unpublished investigations into the GI effects of Herbagut**®**

Herbagut**®** is a proprietary blend of 14 herbal extracts (*Tinospora cordifolia, Hemidesmus indicus, Piper longum Linn, Alpinia galangal, Terminalia chebula Retz, Swertia chirata Buch, Murraya koeniggii, Curcuma longa, and Zingiber officinale*) standardised with total polyphenols of not less than 15% and designed to support GI function. An unpublished in vitro investigation (results included in Additional file 1) demonstrated that Herbagut exhibited antibacterial activity against *Escherichia coli*, *Staphylococcus aureus*, Salmonella typhimurium, Lactobacillus acidophilus, Streptococcus thermophilus, Shigella dysenteriae, Klebsiella oxytoca, Vibrio cholera, and *Helicobacter pylori*. Lower minimum inhibitory concentrations were demonstrated against the unfavourable bacteria, Streptococcus thermophiles, Shigella dysenteriae, Klebsiella oxytoca, and Vibrio cholera. In an unpublished animal study, the laxative effects of Herbagut was examined in rats. Constipation was induced by the daily oral administration of loperamide hydrochloride (3 mg/kg) for six days. Treated rats were orally given Herbagut (50, 75 and 100 mg/kg) once daily for 6 days, after which time 24-h faecal matter was collected. The animals were then sacrificed on day 7 and the number of faecal pellets in the colonic lumen was counted and their mean diameter measured. Herbagut administration significantly alleviated loperamide-induced constipation, as evidenced by an increase in 24-h faecal pellet number and water content discharge, and a reduction in the number and mean diameter of faecal pellets remaining in the colonic lumen compared to control rats. Herbagut at a dose of 75 mg/kg was found to be most effective.

### Study aims

The objective of the present trial was to evaluate the efficacy and tolerability of Herbagut in adults presenting with self-reported unsatisfactory bowel habits. It was hypothesised that its administration over a 4-week period would positively influence several indicators associated with GI function, GI disturbance, and overall quality of life.

## Methods

### Study design

**Fig. 1:**
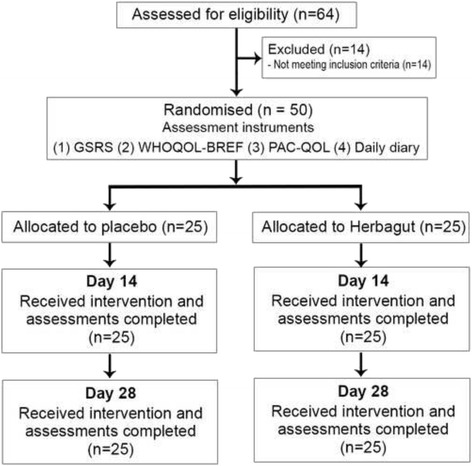
Systematic Illustration of Study design

This study was a 28-day, randomised, double-blind, placebo-controlled trial evaluating the efficacy and tolerability of Herbagut on GI health and its effect on quality of life parameters in adults with self-reported unsatisfactory bowel habits, primarily characterised by chronic constipation. The study protocol was approved by ‘Nagpur Independent Ethics Committee’ and registered with the Clinical Trials Registry- India (CTRI registration number: CTRI/2016/11/007479) with participant recruitment occurring between July 2015 to September 2015. Details of the study design are outlined in Fig. 1.

An a priori power analysis was undertaken to estimate required sample size. We predicted a moderate effect size of 0.8 for the treatment group. Assuming a power of 80% and a type one error rate (alpha) of 5%, the total number of participants to find an effect was estimated as 50. Enrolled participants were assigned to either one of the two study groups (Herbagut or placebo) according to a computer-generated randomisation sheet in a 1:1 ratio. A randomisation list with only the randomisation numbers was provided to the study site for the purpose of enrolling volunteers in the study. The master randomisation list with the details of allocation was kept safely and confidentially with the study sponsor.

Potential participants were screened after being provided with details about the study (both verbally and in writing) and signing an informed consent form. Fifty eligible participants were enrolled in the study as per inclusion and exclusion criteria. Participants attended 4 visits in total to Samvedna Hospital, India, during the study period. During visit 1 (day − 5 to − 1) informed consent documentation, demographic data, medical history, physical and systemic examination, vital parameters including respiratory rate, electrocardiography, chest x-ray, haematology, biochemistry, serology, and urine analysis were performed. In addition, a pregnancy test was undertaken on female participants. On visit 2 (day 0) eligible participants were enrolled into the trial and were randomised into one of two treatment conditions (Herbagut or Placebo). Participants were also asked to complete several self-report measures at baseline, day 14, and day 28, comprising a daily bowel habits diary, World Health Organization Quality of Life – abbreviated version (WHOQOL-BREF), Gastrointestinal Symptom Rating Scale (GSRS), and Patient Assessment of Constipation-Quality of Life (PAC-QOL). Participants were also questioned about tolerability to capsule intake and adverse events. During visit 4 (final visit) unused capsules were also collected and recorded.

### Participants

Patients visiting the Samvedna Hospital in India for a gastroenterology consultation were recruited for this study. Participants were informed about the study, and if agreeable, were assessed by the gastroenterologist or medical officer for eligibility for inclusion in the study.

### Inclusion criteria

Healthy male and female adults aged between 18 to 65 years, a body mass index (BMI) between 20 and 30 kg/m^2^, with self-reported unsatisfactory bowel habits, baseline Bristol Stool Form type 1–2, and GSRS ratings of 2 or less on all questions were eligible for inclusion in this study. Participants were willing to participate in the study and comply with its procedures by signing a written informed consent. Participants also met inclusion criteria if they reported no regular use of fibre supplements the month prior to the screening visit. Participants were willing to discontinue the intake of probiotics, prebiotics, fermented milk products, yoghurt, or laxative use at least 4 weeks prior to, and during the study period. Female participants of child-bearing age were willing to use a reliable method of contraception throughout the study period. Results of a pregnancy test undertaken at screening also needed to be negative.

### Exclusion criteria

Participants were ineligible for the study if they were diagnosed with a gastrointestinal disease (e.g., irritable bowel disorder, Crohn’s disease, Coeliac disease) or were assessed as having severe gastrointestinal complications requiring immediate medical intervention, had an allergy or intolerance to lactose or any other food ingredient, regularly consumed probiotics, fibre supplements, prebiotics, yogurt, or laxatives. Participants were also excluded if any clinically relevant abnormalities were found during initial screening or there was a reported history of abdominal surgery (including gastric bypass or laparoscopic banding). Ongoing use of medications known to affect gut motility, such as prokinetic agents, anti-emetic agents, anxiolytics, antidepressants, narcotic analgesic agents, anticholinergic agents for irritable bowel syndrome, medications for constipation, 5HT_3_ antagonists, anti-diarrheal agents, opiate agents used to treat diarrhoea, nonsteroidal anti-inflammatory drugs, and antibiotics taken during or within 4 weeks of study onset, were also included as ineligibility criteria. People with co-morbid illnesses such as alcohol and other drug dependence, cardiovascular, endocrine, renal, or other chronic disease, were also excluded.

### Interventions

400 mg capsules of Herbagut**®** (Arjuna Natural Extracts Ltd., Aluva, Kerala, India) and matching placebo (roasted rice powder) were used for the intervention and placebo groups, respectively. Participants were instructed to take two capsules, 1-h before bedtime with 250 ml of water. The herbal drug and placebo (roasted rice powder) was filled in ‘0’ size green coloured opaque hard capsules. This prevented bias based on smell, taste and appearance between the herbal drug and placebo. The herbal drug and placebo capsules for each participants was also dispensed in sealed bottles which will also helped maintain blinding.

### Details of study drug

Herbagut**®** is a proprietary blend of 14 herbal extracts containing *Tinospora cordifolia stem* (Guduchi or Amrita), *Hemidesmus indicus rhizomes* (Indian Sarsaparilla), *Piper longum Linn fruit* (Indian Long Pepper), *Alpinia galangal rhizomes* (Thai Ginger), *Terminalia chebula Retz fruit* (Black- or Chebulic Myrobalan), Andrographis panilulata *dried stem and leaves (Swertia chirata Buch)*, *Murraya koeniggii leaves* (Curry Tree*), Curcuma longa rhizome* (Turmeric) and *Zingiber officinale rhizome* (Ginger). The ingredients in Herbagut**®** are blended in equal ratios standardised with total polyphenols of not less than 15% by spectrophotometer, total curcuminoids not less than 6% by high-performance liquid chromatography (HPLC), and total gingerols not less than 0.4% by HPLC.

### Outcome measures

#### Patient daily diary

Participants completed daily monitoring of the following bowel habits:

1. Consistency of bowel movement – this was assessed using the Bristol Stool Form Scale (BSFS). The BSFS is a diagnostic medical tool designed to classify the form of human faeces into seven categories. Type 1 and 2 typifies constipation, type 3 and 4 ideal stools (4 is better), type 5 typifies a precursor to diarrhoea, and types 6 and 7 diarrhoea [18].
2. Ease of bowel movement – this was ascertained from a response to one of six options (manual disimpaction, enema needed, straining needed, normal, urgent without pain, and urgent with pain). Participants were required to choose a single response that best fit their bowel movement characteristics.
3. Evacuation – this was ascertained from a yes or no response to the question, “Did you feel like you emptied your bowels completely?”.

Baseline scores for the Patient Daily Diary were based on ratings made at day 0. However, scores for days 14 and 28 were based on average ratings over the previous 14 days.

#### Gastrointestinal symptom rating scale (GSRS)

The GSRS is a self-report scale that covers 15 gastrointestinal symptoms rated on a 4-point Likert scale, depending on the level of troublesome symptoms over the previous week. Ratings range from 0 (no symptoms) to 3 (severe, incapacitating with inability to perform normal activities). Five subscale scores are calculated for the domains comprising abdominal pain, constipation, diarrhoea, indigestion, and reflux. The GSRS has been shown to have good psychometric properties [19].

#### World Health Organization quality of life – Abbreviated version (WHOQOL-BREF)

WHOQOL-BREF (an abbreviated version of the WHOQOL-100) contains a total of 26 questions, rated on a 5-point Likert scale from 1 (never) to 5 (always) [20]. The WHOQOL-BREF assesses four domains of quality of life comprising physical health (mobility, daily activities, functional capacity, energy, pain, and sleep); psychological health (self-image, negative thoughts, positive attitudes, self-esteem, mentality, learning ability, memory, concentration, religion, and mental status); social relationships (personal relationships, social support, and sex life); and environmental health (financial resources, safety, health and social services, living environment, opportunities to acquire new skills and knowledge, recreation, general environment, and transportation), with higher scores indicating better quality of life. The WHOQOL-BREF has demonstrated good psychometric properties [21].

#### Patient assessment of constipation-quality of life (PAC-QOL)

PAC-QOL is a self-report questionnaire that measures the quality of life of people with constipation. Twenty-eight questions are rated using a 5-point Likert scale ranging from 0 (nothing/ never) to 4 (extremely/ always) with lower scores reflecting better quality of life. Four subscale scores are generated comprising physical discomfort, psychosocial discomfort, worries and concerns, and satisfaction. The PAC-QOL has demonstrated adequate reliability and validity [22].

### Statistical analysis

An independent samples T-test was used to compare demographic and baseline variables across the treatment groups for continuous variables, and Pearson’s Chi-square was used to compare categorical data. The Shapiro-Wilk normality test was conducted to examine normality of within-group data over time. This confirmed that data was not normally distributed, thereby making parametric testing inappropriate (transformations did not normalise data). As a result, within-group changes were analysed using non-parametric tests, comprising either the Wilcoxon signed rank test, Mann-Whitney U-test, or Fisher’s exact test for count data. Between-group differences at differing time points were evaluated with the independent samples Mann-Whitney U-Test. Effect sizes (ES) were calculated for the Mann-Whitney U-test based on the formula, Z^2^ **÷** N-1 (0.1 = small ES, 0.3 = medium ES; 0.5 = large ES). For all the tests, statistical significance was set at *P* < .05 (two-tailed). All data were analysed using SPSS (version 22; IBM, Armonk, NY).

## Results

### Demographic details and baseline data

A total of 50 participants (29 males and 21 females) were enrolled in the study with all volunteers completing the 28-day trial. Data was also collected in full, with no missing data from assessment instruments. Demographic characteristics are shown in Table 1 and indicate that the study population was largely homogeneous, with no statistically significant differences between the groups on demographic characteristics. However, there were statistically significant between-group differences at baseline for WHOQOL-BREF overall quality ratings and baseline PAC-QOL values for physical discomfort, psychosocial discomfort and worries/ concerns (lower scores in the placebo condition).

**Table 1:** Participant baseline demographic characteristics

	Herbagut	Placebo	*p*-value
Female (n)	9	12	.390^b^
Male (n)	16	13	
Age, (mean & SD)	45.72 (8.60)	47.52 (6.31)	.403^a^
Weight (kg), (mean & SD)	60.2 (10.05)	58.6 (9.18)	.569^a^
BMI (mean & SD)	21.60 (1.62)	21.60 (1.45)	.993^a^
Patient Daily Diary			
Type 1 & 2 Bristol Rating	25	25	1.00^b^
Incomplete evacuation (n)	21	23	.384^b^
Bowel movement per week (mean)	1.52	1.32	.298^b^
Stool consistency (mean)	1.88	2.44	.051^b^
GSRS Baseline Scores (mean)			
Abdominal Pain	5.04	5.12	.820^c^
Constipation	5.68	5.20	.133^c^
Diarrhoea	4.96	5.32	.468^c^
Indigestion	7.04	6.28	.106^c^
Reflux	4.12	3.60	.145^c^
WHO-QOL Baseline Scores (mean)			
Overall Quality	4.24	3.76	.041^c^
Social Relationships	46.72	37.92	.099^c^
Environment	38.96	34.64	.234^c^
Psychological	35.60	34.56	.608^c^
Physical Health	40.68	40.28	.976^c^
PAC-QOL Baseline Scores (mean)			
Physical Discomfort	3.09	1.91	< .001^c^
Psychosocial Discomfort	2.76	2.16	< .001^c^
Worries & Concerns	2.92	2.12	< .001^c^
Satisfaction	2.50	2.41	.321^c^

*SD* standard deviation; ^a^Independent samples T-Test; ^b^Pearson Chi-Square test; ^c^Independent Samples Mann-Whitney U-Test

### Patient daily diary

Table 2 details findings from the Patient Daily Diary. In the Herbagut group, weekly bowel movements increased over time (*p* < .001). At baseline, only 12% of participants reported ≥3 bowel movements a week which increased at the end of treatment to 72%. In contrast, there was no change in frequency of bowel movements in participants in the placebo group (*p* = .414).

**Table 2:** Patient Daily Diary changes over 28-day intervention (percentages represent proportion of participants for each measure)

	Herbagut			Placebo		
	Baseline	Day 14	Day 28	Baseline	Day 14	Day 28
≥ 3 BOWEL MOVEMENTS PER WEEK	12%	28%	72%	4%	4%	4%
BRISTOL RATINGS						
Type 1 & 2 (constipation)	100%	36%	24%	100%	60%	52%
Type 3 & 4 (healthy stool)	0%	64%	44%	0%	24%	32%
Type 5 (precursor to diarrhoea)	0%	0%	28%	0%	16%	12%
Type 6 & 7 (diarrhoea)	0%	0%	4%	0%	0%	4%
EASE OF BOWEL MOVEMENTS						
Manual disimpaction	56%	8%	16%	32%	32%	32%
Enema needed	32%	16%	0%	36%	24%	32%
Straining	0%	4%	0%	24%	24%	24%
Normal	0%	56%	76%	4%	8%	4%
Urgent without pain	0%	12%	8%	0%	8%	0%
Urgent with pain	12%	4%	0%	4%	4%	8%
INCOMPLETE EVACUATION	84%	24%	24%	92%	72%	76%

An analysis of change in stool type over time, as measured by the BSFS indicated positive stool changes in both the Herbagut and placebo groups. At baseline, no participants reported ideal stool forms (i.e., type 3 and 4) in the Herbagut group, which increased to 44% at day 28. Positive stool changes also occurred in the placebo group, where rates of healthy stools moved from 0% at baseline to 32% at day 28. A Fisher’s exact test for count data demonstrated significant differences in the frequency of constipation (type 1 and 2) at day 28 between the placebo and Herbagut groups, with lower frequencies in the Herbagut group (*p* = .040). Ease of bowel motions also increased significantly in the Herbagut group, with no participants reporting normal bowel movements at baseline, and 76% at the end of treatment. No change in bowel movements occurred in the placebo group with 4% at baseline and end of treatment reporting normal bowel movements. A Fisher’s exact test for count data demonstrated significant differences in self-reported ease of bowel movement at day 28 between the placebo and Herbagut groups, with greater frequency of normal bowel movements in the Herbagut group (*p* < .001) Finally, self-reported incomplete evacuation decreased significantly in the Herbagut group, with rates decreasing from 84% to 24%, from baseline to treatment completion, respectfully. No significant reductions occurred in the placebo group as rates of incomplete evacuation were 92% at baseline and 76% at day 28. A Fisher’s exact test for count data demonstrated significant differences in the frequency of complete evacuations at day 28 between the placebo and Herbagut groups, with greater frequencies in the Herbagut group (*p* = <.001).

### GSRS

**Fig. 2:**
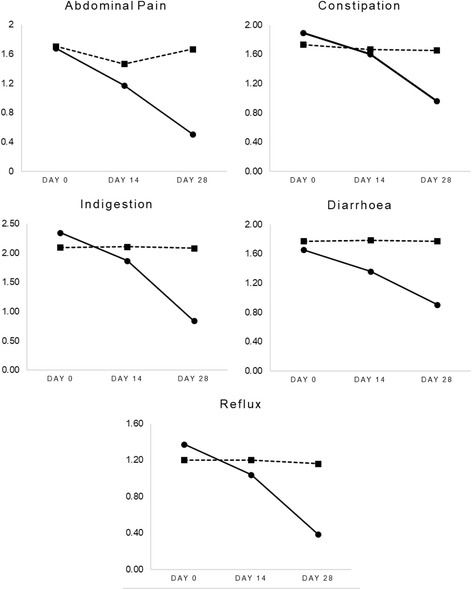
GSRS domain ratings over time.

Results from the GSRS domain ratings over time are presented in Fig. 2. As indicated, ratings for abdominal pain, constipation, diarrhoea, indigestion, and reflux decreased significantly in people taking Herbagut. Abdominal pain ratings decreased by 70%, constipation by 49%, diarrhoea by 45%, indigestion by 64%, and reflux by 72%. However, no significant changes in these corresponding domains were found in people taking the placebo. A Wilcoxon signed-rank test confirmed that GSRS ratings on all domains decreased significantly from baseline to day 28 in the Herbagut group (*p* < .001, for all domains). However, there were no statistically significant changes in the placebo group. As demonstrated in Table 3, an independent samples Mann-Whitney U Test confirmed statistically significant, and large between-group differences on all domain scores at day 28.

**Table 3:** Between group significance values at days 14 and 28 and overall effect sizes (Independent Samples Mann-Whitney U Test)

		*p*-value^a^	Effect size^b^
GSRS			
Abdominal Pain	Day 14	.021	
	Day 28	<.001	0.74 (large)
Constipation	Day 14	.613	
	Day 28	<.001	0.59 (large)
Diarrhoea	Day 14	.008	
	Day 28	<.001	0.61 (large)
Indigestion	Day 14	.216	
	Day 28	<.001	0.73 (large)
Reflux	Day 14	.157	
	Day 28	<.001	0.79 (large)
WHOQOL-BREF			
Social Relationships	Day 14	.005	
	Day 28	<.001	0.48 (medium)
Overall Quality	Day 14	<.001	
	Day 28	<.001	0.55 (large)
Environment health	Day 14	<.001	
	Day 28	<.001	0.78 (large)
Psychological health	Day 14	<.001	
	Day 28	<.001	0.68 (large)
Physical health	Day 14	<.001	
	Day 28	<.001	0.66 (large)
PAC-QOL			
Physical discomfort	Day 14	.004	
	Day 28	<.001	0.66 (large)
Psychosocial discomfort	Day 14	.021	
	Day 28	<.001	0.65 (large)
Worries and concerns	Day 14	.067	
	Day 28	<.001	0.57 (large)
Satisfaction	Day 14	.001	
	Day 28	<.001	0.61 (large)

^a^Mann-Whitney U Test; ^b^effect size of Mann-Whitney U test (Day 0 to Day 28)

### WHOQOL-BREF

**Fig. 3:**
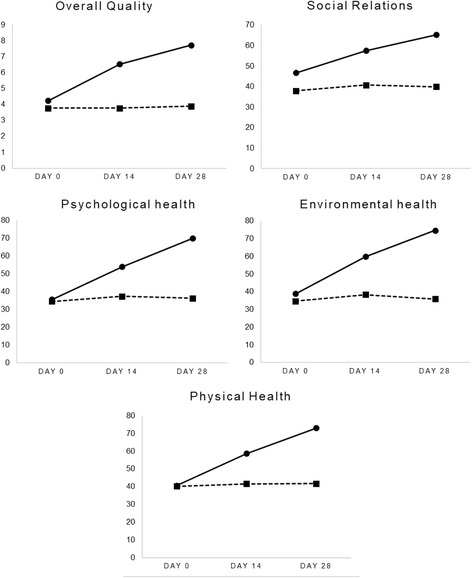
WHOQOL-BREF domain ratings over time.

Ratings for the five domains measured by the WHOQOL-BREF are detailed in Fig. 3. In the Herbagut group, significant rating changes were demonstrated across all domains as measured by an increase of 82% in overall quality of life, 40% in social relations, 92% in environmental health, 94% in psychological health, and 80% in physical health domain ratings. There were non-significant changes in the placebo demonstrated by reductions from 3 to 5% over time in the aforementioned domains. A Wilcoxon signed-rank test confirmed that WHOQOL-BREF ratings on all domains decreased significantly from baseline to day 28 in the Herbagut group (*p* < .001, for all domains). However, there were no statistically significant changes in the placebo group. As demonstrated in Table 3, an independent samples Mann-Whitney U Test confirmed statistically significant between-group differences in all domain scores at day 28. Large effect sizes were calculated across all domain measures except social relationships where the effect size was medium.

### Pac-QOL

**Fig. 4:**
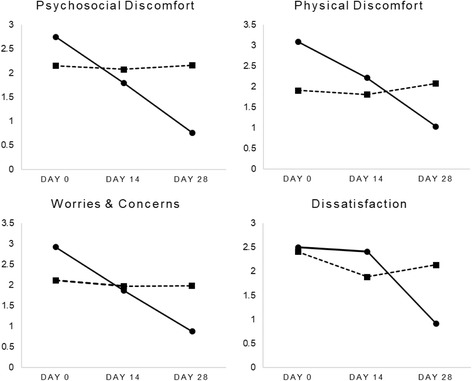
PAC-QOL domain ratings over time.

Results from the PAC-QOL domain scores are presented in Fig. 4. As indicated, ratings for physical discomfort, psychosocial discomfort, worries and concerns, and dissatisfaction significantly improved over time in people taking Herbagut. The satisfaction scales were reversed for ease of interpretation indicating low scores as improvements in satisfaction.

Physical discomfort ratings decreased by 66%, psychosocial discomfort by 72%, and worries and concerns by 70%, while satisfaction scores improved by 64%. However, no statistically significant changes in these corresponding domains were found in people taking a placebo (ratings varied from 0 to 11%). A Wilcoxon signed-rank test confirmed that PAC-QOL ratings on all domains decreased significantly from baseline to day 28 in the Herbagut group (*p* < .001, for all domains). However, there were no statistically significant changes in the placebo group. As demonstrated in Table 3, an independent samples Mann-Whitney U Test confirmed statistically significant and large between-group differences on all domain scores at day 28.

### Adverse events & treatment compliance

Participants were questioned about drug tolerability and adverse events at days 14 and 28. Herbagut was demonstrated to be well-tolerated with no significant adverse events reported by participants. Tolerability of capsule administration was further confirmed by the ability and willingness of all participants to complete the 28-day trial. Compliance with capsule intake was also high with all participants consuming > 90% of allocated capsules (as measured by returned capsule count at day 28).

## Discussion

Findings from this study support the efficacy of Herbagut for the treatment of digestive symptoms and enhancement of overall quality of life in adults with self-reported unsatisfactory bowel habits, suffering primarily from chronic constipation. In this 28-day study, Herbagut was found to be significantly more effective than a placebo as evidenced by improvements in several measures. The GSRS, a self-report measure of digestive symptoms confirmed positive improvements in abdominal pain, constipation, diarrhoea, indigestion, and reflux. Improvements in stool form, as measured by the BSFS, provides additional support for the digestive-enhancing efficacy of Herbagut, as ideal stool forms increased from 24% of participants at baseline to 56% at the end of treatment. Moreover, the frequency of bowel motions increased in participants taking Herbagut, with 12% of participants reporting ≥3 motions per week at baseline, and 76% of participants at the end of treatment. Finally, self-reported incomplete evacuation decreased in the Herbagut group, with rates decreasing from 84% pre-treatment to 24% post-intervention. No such improvements occurred in participants placed on placebo.

An examination of changes in quality of life also confirmed that Herbagut intake is associated with significant and clinically meaningful positive improvements in a range of mood, physical, and social domains. As measured by the WHOQOL-BREF, Herbagut was associated with increased ratings of 82% in overall quality of life, 40% in social relations, 92% in environmental health, 94% in psychological health, and 80% in physical health domains. Similar findings were demonstrated with the PAC-QOL, where ratings for physical discomfort decreased by 66%, psychosocial discomfort by 72%, and worries and concerns by 70%. Life satisfaction ratings also improved by 64%. Although there were significant baseline differences between the two conditions on the PAC-QOL, the large effect sizes demonstrated in this study adds to the clinical significance and validity of study findings. Herbagut was also well-tolerated with no significant adverse effects reported by participants.

The mechanisms behind the efficacy of Herbagut remain uncertain although several possibilities are speculated. Unpublished in vitro investigations into Herbagut have indicated that it exhibits antibacterial activity against *Escherichia coli*, *Staphylococcus aureus*, Salmonella typhimurium, Lactobacillus acidophilus, Streptococcus thermophilus, Shigella dysenteriae, Klebsiella oxytoca, Vibrio cholera, and *Helicobacter pylori*. Lower minimum inhibitory concentrations were demonstrated against the unfavourable bacteria Streptococcus thermophiles, Shigella dysenteriae, Klebsiella oxytoca, and Vibrio cholera. Moreover, several in vitro, animal and human studies have confirmed GI benefits from individual ingredients contained in Herbagut. For example, *Curcuma longa* (Turmeric) has been shown to support gastrointestinal function [23], reduce intestinal inflammation [24], and modify the gut microbiome [25]. *Zingiber officinale* (Ginger) has antibacterial [26], anti-nausea [27], and motility-enhancing effects [28], while *Piper longum* has confirmed antimicrobial and gastro-protective effects [29]. Moreover, *Tinospora cordifolia* contains the digestive enzymes amylase, maltase, and isomaltase [30], and treatment with a formulation containing *Tinospora cordifolia* was shown to have anti-ulcer activity and reduced ethanol-induced gastric mucosal injury in rats [31]. In vitro investigations have also confirmed *Murraya koeniggii* [32] and *Swertia chirata* [33] to have antimicrobial and antibacterial effects. The anti-inflammatory influences from many of these ingredients and evidence suggesting prebiotic-like activities of polyphenols on microbiota [34] may also contribute to the positive GI effects from Herbagut.

### Directions for future research

Findings from this study have confirmed that Herbagut has positive effects on digestive health in adults presenting primarily with symptoms of chronic constipation. However, its efficacy in adults presenting with other FGIDs and GI disorders remains uncertain. Further investigation is therefore essential to determine if Herbagut’s benefits extend to other GI conditions. Medication-induced adverse GI effects is also a commonly encountered problem [35], and an investigation into the efficacy of Herbagut to ameliorate such symptoms may also be fruitful. This is particularly important as medication compliance can be a problem for people experiencing drug-induced GI disturbances [36]. However, before this can be undertaken, the potential interaction of Herbagut with such medications requires consideration.

GI disturbances are commonly associated with a range of other disorders. These include psychiatric disorders [37], neurological disorders such as Alzheimer’s disease [38] and Parkinson’s disease [39], metabolic disorders [40], and several dermatological conditions [41, 42]. Interest in the gut-brain interaction and the potential implications on psychiatric and neurological function is attracting increasing scientific and clinical attention. This is specifically indicated by increasing investigations into, and popularity of, probiotics. In recent meta-analyses, it has been confirmed that probiotics have some therapeutic benefit for the treatment of depression [43, 44]. Herbagut, with its poly-herbal formulation, may be a potential option to enhance both GI and mental function in people suffering from psychiatric conditions. The positive change in the quality of life from the intake of Herbagut, and recent research confirming antidepressant benefits of curcumin [45], one ingredient contained in Herbagut, adds weight to the therapeutic possibility of Herbagut in this area. Herbagut also has the potential to influence imbalances in bacterial ecology and parasitic infection, and this could be another area of investigation.

### Study limitations

Although findings from the study were positive, there are several study design limitations. As already discussed, the efficacy of Herbagut was examined in a population presenting with chronic constipation and no formal GI diagnosis. This limits the generalisability of findings to other populations presenting with divergent GI symptoms. Diagnoses of FSIDs based on Rome classifications will provide greater assessment of the efficacy of Herbagut in more well-defined GI conditions. Participants for this study were only recruited from India, where the intake of many of the herbs and ingredients used in Herbagut may be more commonplace. Its tolerability and efficacy in other populations and cultures, therefore, requires further investigation. Moreover, Herbagut was only used for a comparatively short period of 28-days, and the sample size recruited in this study was also relatively small. It is, therefore, important that future studies be conducted over a longer duration, using larger sample sizes.

Although considered a generally valid indicator of change, only subjective assessments were used in this study to examine symptomatic changes over time. However, some difficulties were encountered with the use of the selected self-report instruments. For example, to assess ease of self-reported bowel movements, participants were required to choose only one response from the available options. This resulted in approximately 40% of respondents describing problems of manual disimpaction at baseline. This is a high number and may be skewed as respondents were only permitted one response. A similar difficulty was encountered with the GSRS where participants were required to answer all questions. However, based on anecdotal reports, rating options for some questions were unsuitable, particularly those assessing stool consistency. For example, on one question the options ranged from ‘normal consistency to watery stools.’ This seemed to artificially raise scores in the diarrhoea domain despite most participants predominantly suffering from constipation. The choice and design of subjective assessments to help assess baseline characteristics and change over time will require careful consideration in future studies. This could be partly overcome by including objective measures of GI health in the future to validate subjective changes. These could include stool examinations of microbial ecology or blood collections to measure inflammatory and other pertinent measures of change. Moreover, because only recordings over a single day was used to form baseline data (rather than the typical 1 to 2 weeks of data) this limits it reliability. In future studies, baseline information collected over a longer period would be preferable.

Finally, the effects of Herbagut, a blend of multiple ingredients on GI symptoms was investigated. This makes it impossible to determine the unique influences of each individual ingredient on GI symptoms and overall quality of life. Although there is research to support the GI-supporting efficacy of most individual components in Herbagut, their exclusive effects on symptomatic change were not specifically investigated in our study. Future animal or in vitro studies analysing each ingredient in isolation may help us to better understand the health-enhancing role of each component.

## Conclusions

The findings from this study provide preliminary support for the digestive-enhancing effects of Herbagut in adults with self-reported unsatisfactory bowel habits, primarily comprised of chronic constipation. Herbagut ingestion over a 28-day period resulted in improvements in several GI symptoms and overall quality of life. Further investigation into this promising poly-herbal blend utilising larger sample sizes and diverse clinical and cultural populations will help us to better understand its health-enhancing efficacy.

## Additional file

Additional file 1: In vitro Antibacterial Activity of Herbagut. (DOCX 14 kb)

## Abbreviations

BMI: Body mass index
BSFS: Bristol Stool Form Scale
CAM: Complementary and alternative medicine
FGIDs: Functional gastrointestinal disorders
GI: Gastrointestinal
GSRS: Gastrointestinal Symptom Rating Scale
HPLC: High-performance liquid chromatography
PAC-QOL: Patient Assessment of Constipation-Quality of Life
WHOQOL-BREF: World Health Organization Quality of Life – abbreviated version
