# Supplementary material for: A poly-herbal blend (Herbagut®) on adults presenting with gastrointestinal complaints: a randomised, double-blind, placebo-controlled study
Source: BMC Complement Altern Med. 2018 Mar 20;18:98. doi: 10.1186/s12906-018-2168-y (PMC5859649; doi:10.1186/s12906-018-2168-y)
Supplement: Supplementary file 1 — In vitro Antibacterial Activity of Herbagut. (DOCX 14 kb) [file 12906_2018_2168_MOESM1_ESM.docx]

In Vitro Antibacterial Activity of Herbagut

In vitro screening protocol

Anti-bacterial activity of Herbagut was assessed in vitro against *Staphylococcus aureus, Streptococcus thermophilus, Lactobacillus acidophilus, Escherichia coli, Vibrio cholera, Shigella dysentriae, Salmonella typhimurium, Klebsiella oxytoca* and *Helicobacter pylori* by resazurin dye reduction method (Sarker et al., 2007). A sterile 96 well plate was prepared and labeled under aseptic conditions. Stock solution of Herbagut was prepared by dissolving 100 mg Herbagut powder in DMSO to get concentration 10 mg/ml. A volume of 100μl of Herbagut solution was pipetted into the first row of the plate. To all other wells 50μl of nutrient broth was added. Serial dilutions were performed using a multichannel pipette to get final concentrations from 1000μg/ml to 7.81μg/ml i.e. (1000, 500, 250, 125, 62.5, 31.25, 15.625 and 7. 8125μg/ml). To each well 10μl of resazurin indicator solution and 30 μl of 3x strength isosensitized broth was added. Finally, 10 μl of microbial suspension (5×10^6^ cfu/mL) was added to each well to achieve a concentration of 5×10^5^ cfu/mL. Each plate was wrapped loosely with cling film to ensure that cultures did not become dehydrated. Each Plate had a set of positive (with organism), negative (without organism) and a standard (ciprofloxacin). The plates were prepared and placed in an incubator at 37 °C for 18–24 h. The colour change was then assessed visually. Any colour changes from purple to pink or colourless were recorded as positive. The lowest concentration at which no colour change occur (absence of growth) was taken as the MIC value.

Results

Herbagut showed antibacterial activity against most of the microorganisms tested in this study. The MIC of Herbagut against *E. coli, Staphylococcus aureus, Salmonella typhimurium, Lactobacillus acidophilus, Streptococcus thermophilus, Shigella dysentriae, Klebseilla oxytoca, Vibrio cholera, Helicobacter pylori* was calculated as 500, 500, 500, 500, 250, 125, 250,125, and 62.5 μg/ml respectively (Table 1). Importantly, the significant antibacterial activity was exhibited against unfavourable bacteria with lower MIC values (62.5-250 μg/ml) in contrast to antibacterial activity observed against favourable bacteria with higher MIC values (250-500 μg/ml).

**Table 1: MIC of Herbagut and standard (ciprofloxacin) against bacteria by microtitre method**

| **S. No.** | **Organism** | **MIC value (μg/ml)** | |
| --- | --- | --- | --- |
|  |  | **Herbagut** | **Standard** |
| 1 | *E. coli* | 500 | 1000 |
| 2 | *Staphylococcus aureus* | 500 | 500 |
| 3 | *Salmonella typhimurium* | 500 | 1000 |
| 4 | *Lactobacillus acidophilus* | 500 | 250 |
| 5 | *Streptococcus thermophilus* | 250 | 31.25 |
| 6 | *Shigella dysentriae* | 125 | 15.125 |
| 7 | *Klebseilla oxytoca* | 250 | 500 |
| 8 | *Vibrio cholera* | 125 | 125 |
| 9 | *Helicobacter pylori* | 62.5 | 15.125 |

References

Sarker SD, Nahar L, Kumarasamy Y. Microtitre plate-based antibacterial assay incorporating resazurin as an indicator of cell growth, and its application in the in vitro antibacterial screening of phytochemicals, Methods. 2007 August; 42(4): 321–324.
